# Quality of Obstetric Care in Public-sector Facilities and Constraints to Implementing Emergency Obstetric Care Services: Evidence from High- and Low-performing Districts of Bangladesh

**DOI:** 10.3329/jhpn.v27i2.3327

**Published:** 2009-04

**Authors:** Iqbal Anwar, Nahid Kalim, Marge Koblinsky

**Affiliations:** Public Health Sciences Division, ICDDR,B, GPO Box 128, Dhaka 1000, Bangladesh

**Keywords:** Emergency obstetric care, Health facilities, Health services, Maternal health, Maternal health services, Obstetric care, Quality of care, Rural health services, Bangladesh

## Abstract

This study explored the quality of obstetric care in public-sector facilities and the constraints to programming comprehensive essential obstetric care (EOC) services in rural areas of Khulna and Sylhet divisions, relatively high- and low-performing areas of Bangladesh respectively. Quality was explored by physically inspecting all public-sector EOC facilities and the constraints through in-depth interviews with public-sector programme managers and service providers. Distribution of the functional EOC facilities satisfied the United Nation's minimum criteria of at least one comprehensive EOC and four basic EOC facilities for every 500,000 people in Khulna but not in Sylhet region. Human-resource constraints were the major barrier for maternal health. Sanctioned posts for nurses were inadequate in rural areas of both the divisions; however, deployment and retention of trained human resources were more problematic in rural areas of Sylhet. Other problems also plagued care, including unavailability of blood in rural settings and lack of use of evidence-based techniques. The overall quality of care was better in the EOC facilities of Khulna division than in Sylhet. ‘Context' of care was also different in these two areas: the population in Sylhet is less literate, more conservative, and faces more geographical and sociocultural barriers in accessing services. As a consequence of both care delivered and the context, more normal vaginal and caesarian-section deliveries were carried out in the public-sector EOC facilities in the Khulna region, with the exception of the medical college hospitals. To improve maternal healthcare, there is a need for a human-resource plan that increases the number of posts in rural areas and ensures availability. All categories of maternal healthcare providers also need training on evidence-based techniques. While the centralized push system of management has its strengths, special strategies for improving the response in the low-performing areas is urgently warranted.

## INTRODUCTION

**Fig. 1.**
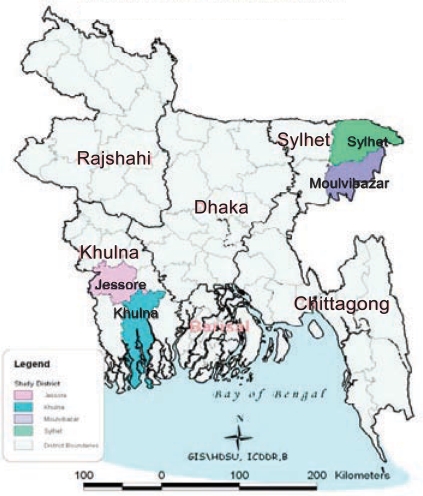
Map of Bangladesh showing study districts

Bangladesh has made significant progress towards achieving the Millennium Development Goal 5 target of 75% reduction in the maternal mortality ratio (MMR) with a very low use of skilled birth attendants, a low caesarean-section rate, and persistent regional variation in the use of maternal healthcare services. The southwest region (Khulna division) performs relatively better while the northeastern Sylhet division lags behind with very low use of maternal health services (Fig. 1 and Table 1) (1).

**Table 1.** Indicator status for maternal and newborn's health in Khulna and Sylhet divisions, Bangladesh

Indicator	Study area		
	All-country	High-performing (Khulna)	Low-performing (Sylhet)
Population∗	138,600,000	5,141,073	4,377,651
Rural	105,100,000	4,116,010	4,048,902
Urban	33,500,000	1,025,063	328,749
Births/year∗	2,877,345	106,729	93,606
Total number of rural UHCs (MoHFW)	409	16	15
Number of rural UHCs targeted to provide comprehensive EOC services (MoHFW)	132	9	5
Women delivered by an SBA (%)†	12.0	16.5	8.8
Deliveries conducted in a health facility (%)†	9.2	12.6	6.1
Population-based caesarian-section rates (%)†	2.7	3.6	1.9
MMR (per 100,000 livebirths) 2001†	322 (95% CI 253-391)	351(95% CI 149-552)	471(95% CI 259-682)
NMR/1,000 livebirths‡	41	47	63
IMR/1,000 livebirths‡	65	66	100
CPR (any method) (%)‡	58.1	63.8	31.8
Total fertility rate∗	3.0	2.8	4.20
Female literacy rate (%)‡	58.8	68.0	47.0

∗Bangladesh Bureau of Statistics, 2005;

^†^ Bangladesh Maternal Health Services and Maternal Mortality Survey 2001;

^‡^ Bangladesh Demographic and Health Survey 2004; CI=Confidence interval; CPR=Contraceptive prevalence rate; EOC=Essential obstetric care; IMR=Infant mortality rate; MMR=Maternal mortality ratio; MoHFW=Ministry of Health and Family Welfare; NMR=Neonatal mortality rate; UHCs=Upazila Health Complexes

This regional contrast in the use of services exists within the broader context of a service programme directed and implemented centrally through the Directorate General of Health Services and of Family Planning of the Ministry of Health and Family Welfare (MoHFW). At the policy level, the strategic approach of the World Health Organization (WHO)—skilled birthcare with back-up support from referral facilities—is well-accepted. Implementation of this approach depends on the existing infrastructure and human resources of the broader health system of the country (2). However, programmes aiming at improving maternal health are not only technical, social interventions are also needed to bring about change (3). The time is right to address the challenges of effective implementation of services at the district level and below (4).

This paper describes the supply side of the safe motherhood programme to understand the reasons for regional variations in the use of maternal health services between Khulna and Sylhet divisions of Bangladesh. We compared the structure, process, and outcome dimensions of quality of care in the public-sector obstetric care facilities in Khulna and Sylhet divisions (5,6). We also reviewed service implementation from the district manager's perspective to understand their constraints in programming comprehensive EOC services in rural areas of the country.

The demand side of the equation, as evidenced by the use of healthcare services, is determined, by and large, by the availability of quality healthcare services and by reduction of more context-specific barriers—both economic and cultural (7). The demand for care in Khulna and Sylhet is explored through responses of women to postpartum haemorrhage and eclampsia (8).

The general objective of the study was to describe the provision of maternity care in two divisions in Bangladesh while the specific objectives were: (a) to explore the quality of care in the public-sector obstetric care facilities in Khulna and Sylhet divisions of the country and (b) to understand the constraints encountered and possible solutions in implementing the comprehensive EOC programme in rural areas of these two divisions.

### Background

In 1994, Bangladesh initiated implementation of a safe motherhood programme using an EOC strategy to address the high MMR. The programme received further impetus in 1998 when a new reform targeted specific delivery sites to be upgraded to provide comprehensive EOC services; these included all medical college hospitals (n=14), all district hospitals (n=59), 64 of 90 Maternal and Child Welfare Centres (MCWCs), and 132 (33% of 409) subdistrict-level Upazila Health Complexes (UHCs). All other rural UHCs and a selected number of union-level Family Welfare Centres (FWCs) were to provide basic EOC services (9). Upgradation meant renovating the existing infrastructure of targeted comprehensive EOC facilities, training the needed human resources, and establishing a separate national reproductive health programme office to ensure supply of required instruments and medicines essential for comprehensive EOC services.

Human resources for health in a district do not depend upon the population but on the number of health facilities and the number of beds. For example, any rural UHC has nine posts for doctors and 11 posts for nurses, irrespective of the catchment population. Of nine sanctioned posts for doctors in a rural UHC, there is one post for a consultant obstetrician and one for a consultant anaesthetist. As graduate medical officers are allowed to provide caesarean section or give general anaesthesia if trained in the respective fields, the new safe motherhood programme organized a one-year training for two medical officers from each targeted comprehensive EOC upazila (one on anaesthesiology and the other on obstetrics and gynaecology) to overcome the existing shortage of specialists. Six-month midwifery training was also organized for two nurses from each targeted UHC. A similar training was organized for the staff of MCWCs: a six-month training on midwifery for Family Welfare Visitors (FWVs) and a one-year specialist training (anaesthesia and obstetrics) for medical officers.

## MATERIALS AND METHODS

The present study reviewed maternal health policies and programmes at the national level through meetings and workshops of stakeholders and through a document review. Operationalization of the maternal health programme was examined in terms of quality and constraints in selected districts in high- and low-performing divisions using both qualitative and quantitative research methods.

### Site selection

In a safe motherhood stakeholders' meeting attended by researchers, policy-makers, programme managers, and development partners in December 2004, several hypotheses were formulated to explain the reduction of MMR in Bangladesh witnessed over the previous decade (10) and the variation in the use of services and of MMR reported by division. To investigate the variation in implementation and demand, the stakeholders selected high- and low-performing divisions on the basis of available data on the MMR (cut-off 400 per 100,000 livebirths) and skilled birth attendance rate (cut-off 15%). Khulna and Sylhet divisions were selected as the high- and low-performing divisions respectively (Fig. 1); specific districts within each division were then selected as representative of the division (Khulna and Jessore districts from Khulna division and Maulvibazar and Sylhet districts from Sylhet division). In the high-performing districts, nine of 16 UHCs had been targeted by the Government to provide comprehensive EOC services while five of 15 rural UHCs were targeted in the low-performing districts (Table 1).

### Data-collection methods

Both qualitative and quantitative data-collection methods were employed to address the research questions. Quantitative data were gathered by physically inspecting each public-sector facility in the selected districts by a two-person team, including one medical doctor. Two monitoring reviews were conducted—one in 2005 and the other one in 2006/2007. The first review collected demographic and human-resource data from all the four study districts and quality-of-care data from only those facilities targeted by the Government to be upgraded as comprehensive EOC facilities (n=24). The second survey was conducted in all the public-sector EOC facilities (basic or comprehensive) in the study districts—medical college hospitals, district hospitals, MCWCs (10-bed hospitals), and rural UHCs (31-bed hospitals) to explore the quality of care on a wider sample of public-health facilities (n=41).

Under the ‘structure' dimension of quality of care, details were collected on the distribution of functioning EOC facilities and human resources relevant for maternal health (sanctioned posts, number posted and present). For the ‘process' dimension, we collected information on availability and functioning of operating theatres, labour rooms, and obstetric wards, including instruments, equipment, essential drugs, and blood. Data on the practice of evidence-based techniques, such as active management of the third stage of labour, use of magnesium sulphate for eclampsia, use of partograph, and infection-prevention practices (11), were also tabulated. These process data were collected using a prepared quality-monitoring checklist developed from the guidelines of United Nations (UN) (12) that were adapted in consultation with members of the professional association—Obstetric and Gynecological Society of Bangladesh. An element was recorded ‘present' when it was found physically in good working condition. An evidence-based technique was practised if there was record of performance within the last three months in service registers. The team also checked the signal functions as detailed in the UN guidelines. The ‘outcome' dimension captured data on performance from these facilities, such as numbers of caesarean sections, normal deliveries, and referrals during the last month and the last one year through review of records (maternity register, operating theatre register, bed-head tickets, case notes, and other relevant data from routine facility-management information systems). Before finalization, the checklist was pretested in two public-sector EOC facilities outside the study districts.

To determine if a facility was a functioning EOC facility, we used criteria based on the UN guidelines (12):

• By definition, basic EOC facilities must have performed each of six signal functions at least once in the previous three months, including parental antibiotics, oxytocics, anticonvulsants, manual removal of retained placenta, and removal of retained products. Assisted vaginal delivery was excluded as an essential criterion for BEOC as it is rarely performed in Bangladesh.
• Comprehensive EOC facilities add caesarean delivery and blood-transfusion performance to this list. In our hands, if blood-bank or blood-storage facilities were not available but blood could be managed from other places, the facility was accepted as a functional comprehensive EOC facility.

Qualitative methods explored supply-side constraints to, and solutions in, programming comprehensive EOC through in-depth interviews with service providers (obstetricians [n=6], anaesthetists [n=2], nurses [n=2], FWVs [n=2]), programme managers (at the subdistrict-level Upazila Health and Family Planning Officers [n=4] and at the district-level Civil Surgeons [n=2]) from the high- and low-performing areas, and one central-level programme manager from the national EOC programme office. Through such interviews, perceptions of managers were gathered on the constraints behind the performance indicators in the high- and low-performing divisions and solutions they envisioned or used for addressing these constraints.

### Data analysis

Quantitative data were analyzed using the EXCEL-2000 and SPSS software (version 10). A standardized summary quality indicator was computed based on principal component analysis (PCA) and factor analysis (FA) methods (13). All quality variables explored (structure, process, and outcome) from the public-sector EOC facilities were used in the PCA and FA model to come up with a summary quality indicator in standardized scale (mean=0 and standard deviation [SD]=1). This summary indicator compared the quality of EOC services by level of facilities and area. This is a relative rather than absolute measure of quality of care to allow comparability by a single indicator that summarizes all the quality variables explored.

Qualitative data on ‘constraints' in programming safe motherhood generated through in-depth interviews were reviewed on an ongoing basis. These data were entered into Atlas-ti 5.0 using a coding system that identified the main themes and concepts. Content analysis was undertaken to identify trends and patterns from the collected information.

Stakeholder meetings were convened to share research findings, to identify and discuss specific programme areas that need priority attention, and to generate recommendations for future programming in maternal healthcare.

## RESULTS

### Distribution of functioning facilities

The initial implementation plan of the MoHFW in 1998 was to upgrade seven of the 16 UHCs in the Khulna region (Khulna and Jessore districts) and five of the 15 UHCs in the Sylhet region (Sylhet and Maulvibazar districts) to function as comprehensive EOC facilities. A further two UHCs in the Khulna region started functioning as comprehensive EOC facilities on their own initiative, and the government target was reset to nine. During the first monitoring review (2005), all the targeted UHCs (9) were functioning as comprehensive EOC facilities in Khulna while, in the Sylhet region, only one of the five targeted UHCs was functioning as a comprehensive EOC facility (Table 1). During 2006/2007, eight of the nine targeted UHCs were functioning as comprehensive EOCs in the high-performing districts while none was so performing in the low-performing districts. At the district level and above, all the targeted comprehensive EOC facilities (medical colleges, district hospitals, and MCWCs) in both the areas were functioning as comprehensive EOC facilities during the three-year study period.

**Fig. 2.**
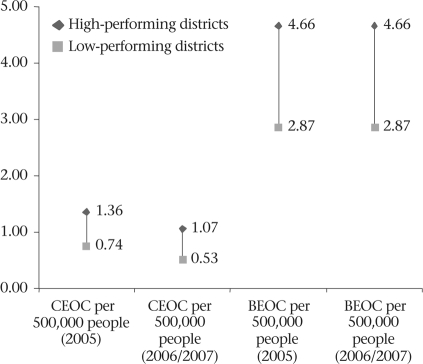
Concentration of functioning EOC facilities for maternal health in high- and low- performing districts, 2005-2007

While the planned implementation of the comprehensive EOC facilities satisfied the minimum UN criteria of at least one comprehensive EOC facility for every 500,000 people (11) in both the areas, the actual distribution of the functional comprehensive EOC facilities only satisfied this criterion in the Khulna region in both 2005 and 2006/2007. The concentration in the Sylhet region was less—about 0.74 in 2005 and 0.53 per 500,000 people in 2005 and 2006/2007 (Fig. 2). A decreasing trend of available comprehensive EOC facilities was observed in both the areas over the study period.

BEOC=Basic essential obstetric care; CEOC=Comprehensive essential obstetric care

Similarly, the concentration of the functioning basic EOC centres in better-performing Khulna satisfied the UN criteria of at least four basic EOC facilities per 500,000 people in both 2005 and 2006/2007. However, in the poor-performing Sylhet region, there were only 2.87 such facilities per 500,000 people during 2005-2006/2007.

### Human resources

The World Health Report 2005 stipulates the need for four doctors and 20 midwives for every 3,500 births (14) while the World Health Report 2006 recommends a minimum of 2.28 professional care providers per 1,000 people to achieve 80% skilled attendance at birth (15). Human resources for maternal healthcare include nurse-midwives and qualified doctors (MBBS and consultants). ‘Sanctioned posts’ mean the number of posts stipulated by the central government; ‘number posted' is the number of sanctioned posts for which personnel were deployed; and the ‘number available' means the number found during the day of visit. In Bangladesh, the plan behind the sanctioned number of posts of human resources is based on the number of health facilities in rural areas and the number of beds in hospitals (district hospitals and medical college hospitals) in urban areas. Health facilities are established on the basis of administrative units, e.g. districts, upazilas, and not on the basis of population of the area.

In the urban areas of both Khulna and Sylhet, the plan was adequate for maternal care for both doctors (7% and 1.2% sanctioned doctors needed respectively for maternal health) and nurses (35% and 9% sanctioned nurses needed respectively). The human resources plan, however, was inadequate in the rural areas of both high- and low-performing areas for maternal healthcare: over 40% of sanctioned posts for all doctors (not just those involved in maternal care), and over 250% of existing posts of nurses are needed (Table 2).

**Table 2.** Adequacy of human resources: sanctioned posts and availability of nurses and doctors against requirement for maternal health, 2005

Study area	No. of sanctioned posts for all doctors	No. of doctors posted	No. of doctors available on the day of visit	No. of doctors for maternal care required as per WHO criteria	No. sanctioned posts for all nurses	No. of nurses posted	No. of nurses available on the day of visit	No. required for maternal care as per WHO criteria
High-performing								
Urban	352	291	294	24	341	338	365	122
Rural	199	146	119	98	181	175	176	488
Total	551	437	413	122	522	513	541	610
Low-performing								
Urban	637	553	552	08	412	310	317	39
Rural	212	128	72	96	156	175	85	480
Total	849	681	624	104	568	485	402	519

WHO=World Health Organization

When one looks at adequacy in terms of actual availability of staff on the day of visit, the picture was more grim, especially in the rural areas where more than double the numbers of nurses is needed just for maternal care in the high-performing areas and five times the number in the low-performing areas. More than three-quarters of all doctors available would be needed for maternal healthcare alone in the high-performing areas and over 125% of available doctors in the low-performing areas.

**Fig. 3.**
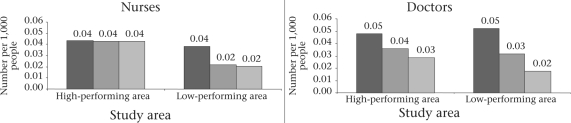
Availability of public-sector doctors and nurses in rural areas: concentration per 1,000 people in high- and low-performing urban and rural areas, 2005

**Fig. 4.**
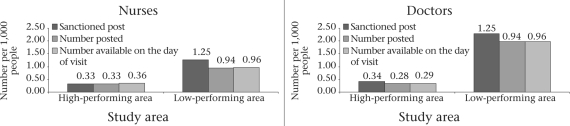
Availability of public nurses and doctors in urban areas: concentration per 1,000 people in high- and low-performing districts, 2005

Our study revealed that human resources for maternal healthcare were considerably more in urban compared to rural areas—more in terms of the plan and actual availability (Fig. 3 and 4). Sanctioned posts in the urban areas were at a level of 0.34 doctors per 1,000 people in Khulna and 1.94 doctors per 1,000 people in Sylhet; in the rural areas of both Khulna and Sylhet, the plan is for 0.05 doctors per 1,000 people. In the urban areas of both Khulna and Sylhet districts, respectively, 83% and 88% of sanctioned posts for qualified doctors were filled and available on the day of visit during 2005.

The WHO criteria of human resources (as of the World Health Report 2006 [15]) were satisfied only in the urban areas of Sylhet division and for nowhere else. This higher concentration of human resources in the urban areas of Sylhet was due to one large, well-established medical college hospital in Sylhet city. If we remove the medical college from the analysis, the overall picture of human resources in the urban areas of the Sylhet region is poor: there are only 34 sanctioned posts of doctors (0.10 sanctioned posts of doctors per 1,000 people).

In the urban areas, about 100% of the sanctioned posts of nurses were filled and available in the high-performing facilities in Khulna but only about three-quarters of sanctioned posts were filled and found available in the facilities in Sylhet. Specifically, the sanctioned posts of nurses and their availability were 0.33 and 0.36 per 1,000 people in urban Khulna and 1.25 and 0.96 per 1,000 people in urban Sylhet respectively (Fig. 3 and 4).

In the rural areas, the sanctioned posts, the proportion of positions filled, and the number of doctors and nurses available were far lower per 1,000 people than in urban areas. In the high-performing rural health facilities, more than 60% of doctors and 100% of nurses were available whereas, in the low-performing areas, only one-third of doctors and half of the nurses in the sanctioned posts were available on the day of visit. Figure 3 and 4 show that the number of sanctioned posts for both doctors and nurses was similar (doctors: 0.05 per 1,000 and nurses 0.04 per 1,000 people) in the rural areas of both high- and low-performing areas. However, their availability was much lower in the low-performing areas than in the high-performing areas (doctors 0.02 and 0.03 per 1,000 people and nurses 0.02 and 0.04 per 1,000 people respectively). The ratio of available qualified doctor to population was 1:34,600 for the rural areas of the high-performing districts versus 1:59,900 in the rural areas in the low-performing districts. The ratio of nurses to population in rural areas was 1:23,400 in the high-performing areas and 1: 66,300 in the low-performing areas.

The availability of specialists—both obstetricians and anaesthesiologists—and trained medical officers to perform surgery in rural areas was a major supply-side barrier for the provision of services. In the rural UHCs, most posts of consultant obstetrician and of consultant anaesthetist were vacant or were filled by medical officers without specialist training. All sanctioned posts of consultant anaesthetist in the five targeted UHCs were vacant in the low-performing districts while only three of nine were filled up in the high-performing districts. Similarly, two of five one-year trained anaesthetists were available in the low-performing areas and six of nine in the high-performing areas. Both anaesthetist and surgeon are needed to perform caesarean sections; such a team was present in all the nine targeted rural comprehensive EOC facilities in the high-performing districts but only in one such targeted facility in the low-performing districts in 2005 (Table 3).

**Table 3.** Availability of obstetrician, anaesthetist, and trained nurses on EOC in targeted rural comprehensive EOCs of high- and low-performing districts, 2005

Human resources for comprehensive EOC	High-performing area (9 UHCs)		Low-performing area (5 UHCs)	
	No. of sanctioned posts and no. trained	No. posted and available	No. of sanctioned posts and no. trained	No. posted and available
Consultant (gynaecologist-obstetrician)	9	7	5	2
Consultant (anaesthetist)	9	3	5	0
One-year trained obstetrician	9	4	5	2
One-year trained anaesthetist	9	6	5	2
A pair of anaesthetist and surgeon	9	9	5	1
EOC-trained nurses	24	20	11	6

EOC=Essential obstetric care; UHCs=Upazila Health Complexes

The human-resource constraints go far beyond the numbers planned/sanctioned and trained. There are serious problems in deployment and retention regarding human resources for maternal health, particularly in rural areas of the country. Programme managers and service providers in both high- and low-performing areas recognized this problem, although its magnitude differed by area.

Many respondents claimed that they could not provide comprehensive EOC services from the targeted UHCs due to the unavailability of a pair of specialists (obstetrician and anaesthetist). One subdistrict manager from Sylhet area stated:

We are not providing any comprehensive EOC service from this UHC, although this is the only targeted rural facility in the district to provide comprehensive EOC. The unavailability of ‘a pair' is the reason. In our UHC, we have a consultant obstetrician but no anaesthetist while, in another UHC, they have one trained anaesthetist but no obstetrician. As a consequence, none of these two UHCs was able to provide comprehensive EOC services. If the authority could maintain ‘the pair' in any one of these two UHCs, at least one could function effectively as a comprehensive EOC facility. It is very much possible to find a way out if we had good management practices at all levels.

One UHC manager from Sylhet area remarked:

To provide comprehensive EOC service, if an untrained medical officer is posted against that of a consultant obstetrician and a cardiologist against the post of an anaesthetist, how is it possible to provide comprehensive EOC services from that facility? What is the use of deploying them?

All the managers from both Khulna and Sylhet areas mentioned problems with the availability of anaesthetists—both in terms of numbers and absenteeism. EOC services are supposed to be available 24 hours a day, seven days a week. However, in many cases, one of the pair lives in the nearby district town and comes to work only once or twice a week. One nurse reported from a UHC in a high-performing area that consultants are called when there is an emergency operation. She remarked about the situation thus:

The obstetrician in our health centre stays here round-the-clock but the anaesthetist does not. In the case of emergency operation at night, the surgeon herself, being previously guided by the anaesthetist, provides anaesthesia for the patient. The anaesthetist usually comes in the morning. He seldom comes at night even if he is called in.

Another anaesthetist from a low-performing area talked about the shortage of anaesthetists at the district-level hospital, stating:

There are two posts of anaesthetist in this district hospital. One post is lying vacant. As a result, it is quite burdensome for me to remain on duty round-the-clock but I have no way left as patients come at any time of the day. This creates a misunderstanding between the surgeons and me (anaesthetist) because I cannot attend all of their calls on time. The number of surgeons is quite a lot in this hospital for only one anaesthetist. It hampers the congenial working environment.

Poor salaries and lack of family amenities in rural areas drive absenteeism. One consultant obstetrician from a high-performing area remarked:

Now-a-days it is very difficult to bear all family expenses with the poor salary of this job. We could stay and provide service round-the-clock if we could have some incentives from the Government. It is not possible to stay there with my family as there is no good school and no opportunity for private practice. For this reason, my family stays in the city, and if I am to stay here, I will have to double the living cost. Considering the facts, I also stay with my family in the city.

In Bangladesh, public-sector physicians are allowed private practice after office hours, and given the poor salary structure in the public sector, they do so. Also doctors trained in EOC do not receive any extra benefit or remuneration for performing EOC. Consequently, doctors are not interested to work in an area where there is a little opportunity for private practice. The rural areas in general are less lucrative for private practice than urban areas, and this is true for the low-performing areas than for the high-performing areas. However, doctors posted in areas where they have a good private practice do not want to get transferred. One obstetrician from a high-performing area said:

I have been posted in this remote area for long 11 years. I had an offer of transfer, which I refused. I have earned fame here, and I can go on with my private practice after office hours. For this reason, it is better for me to stay even in a remote place, as it will be difficult to have such popularity in a new place.

Lack of promotion possibilities and mismanagement in posting and transfer decrease motivation of service providers, as one obstetrician from a low- performing area remarked:

We could work in the remote places if we had a possibility of coming to a better place after two years of service. However, as the management level never takes such an initiative, we lose enthusiasm to work competently. The real picture is that, if someone is posted in such an area, usually he has to be there for quite a long time if he is powerless or not associated with unholy alliances. This is undoubtedly a drawback for providing EOC service in rural areas.

As the public-health system is not decentra-lized, management authority at the local level is non-existent. One manager from a low-performing upazilla mentioned:

I am not in a position to punish someone for irregularities. I can inform my higher authorities about this in writing. And only they can take proper punitive measures but the fact is that all non-residential doctors are politically powerful and personally on good terms with high officials. Why should the professionals stay at their place of posting where there is no accountabi-lity? In addition, they have their clinics or have some relations with some clinics, and they earn a lot from there. Still, they do not resign from their government job as they need to use the label to earn more.

All postings and transfers of specialists are in the hands of the Ministry while those for medical officers trained in EOC are with the Directorate General of Health Services and of Family Planning; in actuality, however, there is an interference from outside the Ministry. A manager in the EOC programme office under the Directorate General of Health Services describes the situation as follows:

In the case of posting of doctors, political interference is the major hindrance. Often a professional organization controls posting and transfer bypassing the formal line-authority. As a result, those who are politically motivated or maintain a good liaison with unionists can easily avoid rural posting. Also sometimes, it happens that doctors posted in remote areas subsequently manage a transfer or deputation to better/urban areas by lobbying with local politicians or with personal lobbying with the authority. As a result, the functionality of EOC in remote rural areas remains the number one problem in the context of Bangladesh with existing management-organization culture.

Another UHC manager of a low-performing area was concerned about the shortage of nurses. In the low-performing areas, posts of nurses remain vacant for years. Those who are available are on shifts to cover 24 hours a day, 7 days a week. The UHC manager stated:

We have been in great difficulties with the shortage of nurses. We are badly in need of two more nurses trained in EOC as the delivery rate here is very high. We conduct almost 100 deliveries a month. Sometimes, 2 or 3 delivery cases come at a time. Only one nurse could be available at that time. Should she manage only the delivery cases or all the other patients? In these circumstances, we take help from a woman sweeper which is unethical, although this is the reality.

### Instruments/equipment and medicines for maternal health

Most equipment and instruments required to organize EOC services are available in all the EOC facilities at both district and upazila levels. However, the situation is a little better in the EOC facilities of the high-performing areas than in the low-performing areas, with the exception of generators and sterile delivery-sets which were found to be more available in the latter (Table 4).

**Table 4.** Availability of logistics in the public sector

Indicator (item available in working condition)	High-performing area (n=21) (%)	Low-performing area (n=20) (%)	All areas (n=41) (%)
Ambulance	95.2	90.0	92.7
Trolley/stretcher	90.5	90.0	90.2
Emergency drugs and IV solution	66.7	90.0	78.0
Sterile gloves	90.5	85.0	87.8
Labour-table with stirrup	90.5	75.0	82.9
Sterilized delivery-set	90.5	100.0	95.1
Generator	71.4	95.0	82.9
Vacuum extractor	57.1	50.0	53.75
Autoclave machine	85.7	75.0	80.5
Sucker machine	100.0	85.0	92.7
Baby-weighing machine	95.2	80.0	87.8
Magnesium sulphate (MgSo_4_)	47.6	25.0	36.6
Oxytocin injections	66.7	50.0	58.5
Three sets of caesarean-section instrument	61.9	50.0	56.1
OT light with spare bulbs	61.9	55.0	58.5
OT table	76.2	65.0	70.7
Anaesthesia machine	61.9	50.0	56.1
On-call staff to perform emergency operation within 30 minutes	76.2	45.0	61.0
Separate post-operative room	66.7	50.0	58.5

IV=Intravenous; OT=Operating theatre

The allocations, for medicines and other supplies for each health facility are fixed at the central level, based on the number of beds per facility. However, instruments, medicines, anaesthetic agents, intravenous fluids, and parenteral antibiotics specific for maternal health are provided from the central programme office on top of the usual budgetary allocations.

The overall supply of medicines was inadequate across all the facilities compared to patient-load. Patients invariably have to buy some medicines when they use public-sector maternal healthcare services, particularly for obstetric surgeries. One obstetrician from Khulna area remarked:

We are getting supply of medicines from the Government; however, it is not sufficient at all. Although the UHCs have 31 beds each, the bed- occupancy rate, on average, is 150%. If it is the scenario, how could we provide adequate drugs to our patients? Due to this inadequate supply of drugs, we have to face problem with patients. When they (patients) ask for drugs, we often tell them to buy drugs from outside. Therefore, they always think that we (management) are stealing medicines from store. This is a very wrong perception of our patients. Although I never blame them as they never know the actual situation of our budgetary allocation for drugs. Sometimes, patients come with false complaints, such as headache and ask for painkiller. We understand that it is a false complaint but we are bound to provide them medicine; otherwise, they usually make serious problem inside the hospital. So, I must say, instead of stealing, some misuse is happening.

For a functional comprehensive EOC, there should be at least two autoclave machines. In one targeted UHC, one autoclave machine was found to take two hours to sterilize instruments. One respondent mentioned that, if a complicated delivery case comes immediately after one caesarean section, she is likely to be referred to another hospital for operation due to the lack of available sterile instruments.

In rural areas, the irregular supply of electricity is a major barrier to providing comprehensive EOC services efficiently. To overcome this problem, each UHC has a power-generator but the supply of fuel for the generators is often insufficient. One obstetrician from a low-performing area remarked:

It becomes difficult on our part to conduct emergency operation when there is no electricity. We have made local arrangements ourselves to overcome the crisis. Usually, we ask the relatives of patients to provide one litre of fuel costing Tk 42 to conduct one operation without interruption in the case of power failure, and they take it easily.

### Evidence-based techniques for maternal health

**Fig. 5.**
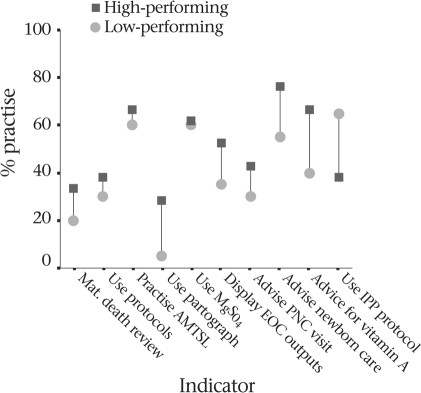
Practice of evidence-based techniques for maternal health in study area

Evidence-based practices were rare in the public-sector facilities of both the areas. The use of magnesium sulphate for eclampsia was about 60% in both the areas while partographs for prolonged labour were used in less than one-third of facilities in the high-performing areas and 5% in facilities in the low-performing areas. Similarly, use of protocols for the management of obstetric complications (38% vs 30%) and display of EOC outputs (52% vs 36%) were a little more available and practised in Khulna areas; even so their overall use was low (Fig. 5). A few places carry out maternal death reviews—only one-third of the EOC facilities in Khulna. Active management of the third stage of labour was practised in nearly two-thirds of the facilities in both Sylhet and Khulna areas. However, infection-prevention protocols were better practised in facilities of the Sylhet region.

AMTSL=Active management of third stage of labour; EOC=Essential obstetric care; IPP=Infection-prevention practices; Mat=Maternal; PNC=Postnatal care

### Blood transfusion

In Bangladesh, there is no blood-bank below the district level. The number of blood-banks is nine in Sylhet (one per 523,000 people) and 11 in Khulna (one for 468,000 people). All these blood-banks are located in urban areas. In most rural facilities, there is no arrangement of blood-grouping and cross-matching. Availability for blood-grouping and cross-matching facilities was lower in Sylhet (20%) than in Khulna (48%). However, blood transfusion had occurred in most rural facilities in both the areas in the last month as blood can be managed from nearby district-level facilities (public or private). Only 55% (11 of 20) of the government facilities had a microscope in Sylhet while nearly all facilities had it in Khulna (Table 5). In both the areas, some UHC patients must go to private diagnostic centres for blood-grouping and cross-matching. At the MCWCs, there was no post for a laboratory technician making blood transfusion difficult during emergency obstetric surgeries, although they are still carried out.

**Table 5.** Availability of blood-transfusion facilities in the EOC facilities

Indicator	High-performing area (n=21) (%)	Low-performing area (n=20) (%)	All areas (n=41) (%)
Availability of blood-grouping and cross-matching facility	47.6	20.0	34.1
Blood-collection bags	14.3	5.0	9.8
Storage facility	23.8	10.0	17.1
Microscope	90.5	55.0	73.2
Refrigerator	57.1	40.0	48.8
Register for recording events	52.4	45.0	48.8
On-call laboratory technician	57.1	40.0	48.8
Blood transfused in obstetric emergencies in the last one month	76.2	95.0	85.4
Voluntary donor list	19.0	5.0	12.2

EOC=Essential obstetric are

### Context of care

Our qualitative exercise revealed that the context of care was different in Sylhet from Khulna. In Sylhet, density of population is lower, and distance between upazila and district town is more compared to Khulna. As a result, recipients of services have to cover more distance to access EOC services. More importantly, there are potential cultural barriers in the Sylhet region: female literacy is lower, people are more conservative, and the religious groups very often influence women not to go outside homes. Moreover, there is no organized middle class as they are obvious in Khulna. These demand-side barriers contributed to the reduced accessibility to and use of maternity-care services.

### Outputs from public-sector EOC facilities

**Fig. 6.**
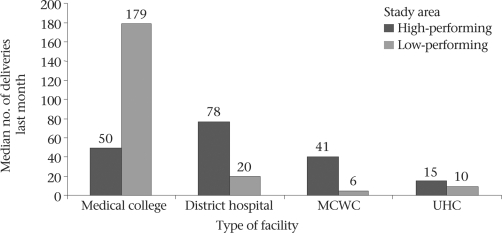
Median number of deliveries conducted in the last month in different types of EOC facilities by study districts, 2006-2007

**Fig. 7.**
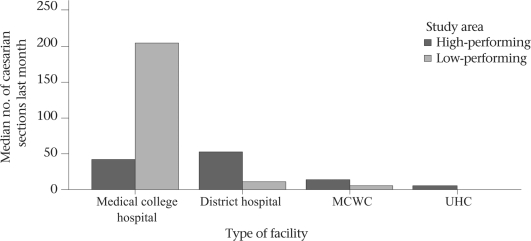
Number of caesarean sections conducted in different public-sector comprehensive EOC facilities in the last month in high-and low-performing districts of Bangladesh, 2005

Most peripheral public facilities in the Khulna region conducted more deliveries in the last month than those in the Sylhet region, with the exception of the medical college hospitals. For example, 15 deliveries were conducted in each UHC, on average, during the last month in the Khulna region, 41 in the MCWCs, and 78 in the district hospitals. In Sylhet, these rates were 10, 6, and 20 respectively (Fig. 6). A similar pattern was observed for deliveries by caesarean section. On average, seven caesarean sections were conducted per month from targeted UHCs providing comprehensive EOC in the high-performing areas while the rate was one per month in a low-performing rural comprehensive EOC facility. Similarly, there were 52 and 14 caesarean sections per month in the district hospitals and MCWCs in Khulna versus 11 and 5 per month in Sylhet (Fig. 7). The exception was at the medical college level. All outputs were much higher in the Sylhet Medical College than those in the Khulna Medical College.

MCWC=Maternal and Child Welfare Centre; UHC=Upazila Health Complex

EOC=Essential obstetric care; MCWC=Maternal and Child Welfare Centre; UHC=Upazila Health Complex

### Summary quality indicator

**Fig. 8.**
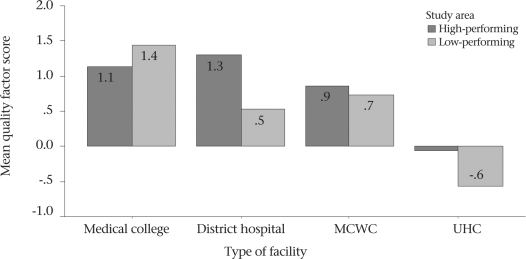
Summary quality score at different EOC facilities by study area

Using a summary quality indicator, the overall quality of care was found to be poorer in the public-sector EOC facilities of Sylhet areas than those facilities of the Khulna region, except the medical college hospital (Fig. 8). Facility-wise, the medical college hospitals were the best, followed by district hospitals and MCWCs; rural UHCs were the worst in both the areas. All the public-sector EOC facilities were functioning better in Khulna than their counterparts in Sylhet, except Sylhet Medical College Hospital—the only place in Sylhet that offered consistent and quality care.

EOC=Emergency obstetric care; MCWC=Maternal and Child Welfare Centre; UHC=Upazila Health Complex

## DISCUSSION

The quality of maternal health services, as measured by structure, process, and outcome, was relatively better in the Khulna region than in Sylhet, although the use of services was low in both the areas. In Khulna, the number of facilities for comprehensive EOC and basic EOC services per 500,000 people met the WHO criteria; the planned levels of staff were available at higher levels in terms of specialists and trained (MBBS) obstetricians and anaesthetists, and nurses trained in EOC; more instruments and equipment were available; and there was more access to blood. This resulted in more normal deliveries and caesarean sections performed from peripheral EOC facilities of the Khulna region.

A limitation of the study was that we did not explore the skills of maternal healthcare-providers in either area. Barring considerations of skills, however, the sheer lack of trained human resources to provide comprehensive EOC is the greatest challenge in rural areas, especially of the Sylhet region.

The plan of the Government for maternal healthcare for rural Bangladesh is insufficient to address this problem of healthcare professionals. This is true for both Khulna and Sylhet but more so for the latter. The plan of the Government calls for about one-tenth the personnel to population ratio in rural areas than in urban areas: this level of approximately 1 per 10,000 is well below the WHO standard of 2.28 professional care providers per 1,000 people to achieve 80% coverage.

Even at the low levels planned, deployment and retention of care providers, particularly the pair of specialists and nurses, in rural facilities, is the major problem for the supply-side. A pair, including a trained obstetrician and anaesthetist (consultant or EOC-trained*),* is needed in each targeted rural comprehensive EOC facility. Yet, trained personnel leave rural postings or, in some cases, do not even join in their posting place. Having only one in the pair is insufficient to implement comprehensive EOC. Both medical officers and consultants trained in EOC manage to avoid serving in rural facilities by acquiring deputation to work at a district hospital or at a medical college hospital (both in urban areas), or they just remain absent in their place of posting. National-level management is grossly unsuccessful in keeping a pair (anaesthetist and obstetrician) in rural designated comprehensive EOC facilities—a symptom of weak governance and inadequate stewardship (16,2). Results of our study suggest that political commitment for maternal health is not just inadequate, it is counter-productive: politicians and trade unions often support poorly-motivated trained professionals to leave rural postings—a threat to the real health system and the functioning of comprehensive EOC facilities in rural areas. Interestingly, some consultant obstetricians and anaesthetists may prefer a posting in UHCs not providing comprehensive EOC services to that in targeted comprehensive EOC facilities so that they can have more time for private practice in a nearby town.

Anaesthetists present a special challenge. The total number of anaesthetists in the country is far less than the number of other types of specialists, and they are rarely available in rural areas, particularly in the low-performing districts. Hence, the challenge is not just deployment or retention but production as well. Medical graduates are reluctant to study anaesthesiology as their work is dependant upon surgeons. The posting plan is also a challenge: If only one anaesthetist is posted in a big hospital, s/he cannot provide day-long service if there is excessive caseload for operative surgeries. Moreover, if the only anaesthetist is sick or on leave, it becomes impossible to provide comprehensive EOC services even from district hospitals.

While these challenges affect the implementation of EmOC in rural areas throughout the country, Sylhet faces particular challenges. Only one of the five targeted facilities was providing comprehensive EOC services in 2005 and none in 2006/2007. Several factors contribute to the poorer human resource scenario of Sylhet, especifically unavailability of an obstetrician and an anaesthetist, including that the rural areas of Sylhet are less lucrative for private practice. Although it can be a conflict of interest in serving the poor (17,18), private practice, allowed by the Government, for public-sector physicians is a major motivating force for doctors and specialists in Bangladesh where the salary structure is poor and career prospects are ill-defined. Demand for services is less in Sylhet: people in this area are less educated and more conservative, mobility of women is restricted, rural facilities are more remote from district towns meaning patients must travel further to access EOC services. The local language of Sylhet is different from the rest of Bangladesh, and most public healthcare providers do not speak the local language as they come from other parts of the country. Moreover, there is no organized middle class in this region: there are only two classes—rich and poor. The rich prefer private or higher-level government services from Sylhet town while the poorest either remain at home or use informal providers for EOC services. As a result, both public and private sectors in rural Sylhet are not growing.

Constraints with nursing professionals are also serious in the Sylhet region. Most nurses employed in Sylhet are non-local while, in the high-performing districts, most are from the same districts. Non-local nurses typically strive to get transferred to an area close to their permanent residential address. Due to the conservativeness in Sylhet, women are reluctant to adopt nursing as their profession. Muslims face religious barriers as they think that female nurses will have to provide care for male patients, which they cannot accept. This is a cultural barrier deeply rooted in societal values and norms. Increasing the number of posts in rural settings and, more importantly, raising the dignity and status of nursing professionals to attract newcomers in this profession are much needed (19,20).

Authority within the Bangladesh health system remains highly centralized. However, our field experience suggests that the governance (management capacity) of the district and divisional health system in Sylhet is weaker than that in Khulna. Sylhet is a relatively newly-established division compared to the older Khulna division where governance and stewardship are better. Although the formal plans for centrally-directed maternal healthcare inputs are identical for both Khulna and Sylhet, the implementation of designed interventions and the use of services are better in Khulna due to the comparatively better response of the local health system and more engaged communities due to higher female literacy, communications systems (roads), higher density of population, a higher social status in terms of mobility of women, and fewer obstacles from fundamental religious groups; however, problems are there as well.

The Government attempts to rectify the inadequacy of specialists by increasing their numbers through one-year training of medical officers on anaesthesia or obstetrics and two-year bonding for rural service have failed to overcome the human resource barrier, particularly for Sylhet. Behind absenteeism and non-residential status of doctors is the sheer lack of motivation of professionals to remain in rural posts. Most respondents (both from Khulna and Sylhet) reported that the poor salary, uncertainty of promotion, absence of uniformity in application of existing rules and regulations in posting, transfer, and promotion are the root causes of professional de-motivation. The Millennium Project assumed that salaries would need to double if the Millennium Development Goals were to be achieved (21). Under the present policy structure of the Government, it is difficult to increase the salary of a special group of public servants. However, it is possible to give a special ‘bonus' or a ‘benefit package' for rural postings of specialists—an incentive that has worked in other settings (22,23). Trials with other strategies to increase the concentration of rural healthcare providers could help. In Gujarat, India, for example, they are experimenting with public-private partnerships to overcome such human resource barriers in rural areas by contracting out maternity-care services to private-sector organizations (24). Preliminary results are encouraging. Improving amenities in rural areas, such as better schooling for children, are also needed, requiring multisectoral involvement; however, this will take time and require improved socioeconomic conditions of the country.

Given the ‘push-system' of the Government's health service, supplies and logistics (medicines, equipment, and instruments) should be uniform in both the study areas. However, facilities in Khulna were found to be relatively better-equipped with supplies (medicines, equipment, and instruments) essential for maternal health, primarily because district authorities have instituted their own ‘pull-system' due to more demand for services. This is one area where local dynamism and responsiveness to demand has paid off—a lesson for all district managers.

Unavailability of blood in rural areas is another major supply-problem for EmOC as it is needed to manage the number one maternal killer—haemorrhage. There is no blood-bank in rural areas of the country, and the number is far less in Sylhet than in Khulna, although more blood transfusions were reported from the Sylhet region. Blood-grouping and cross-matching facilities and the technicians are absent in some targeted UHCs. Eren so blood can be managed even in absence of a blood-bank or without laboratory facilities. When blood is needed, local service providers with the help of attendants of patients manage blood from nearby district towns often with help of private-sector facilities. Very recently, in collaboration with the United Nations Children's Fund/United Nations Development Programme, the Government has begun to establish a network of secure blood-banks in rural areas in response to AIDS but the need continues on an urgent basis for survival of women during delivery.

The results of the lack of sustained quality infrastructure to provide maternal healthcare in low-performing areas are not surprising. A similar situation prevails for other areas of health, in Sylhet, such as child health, family planning, and HIV/AIDS. Both care and context were comparatively better in the Khulna region, including the organization of EOC services, distribution and functionality of facilities, and availability of trained care providers. The market for private practice was also better in Khulna than in Sylhet, a reason why doctors at the UHC level remained at their rural postings in Khulna. All these add up to a relatively-better status of the maternal health indicators in Khulna.

### Recommendations

On the basis of the findings of our study, we recommend that the policy-planners give special attention to certain programmatic and contextual aspects to achieve the target for MDG 5.

- Human resource constraints are the major barrier to achieving maternal health goals. There is need for a human resource plan that increases the number of posts in rural areas and ensures deployment and retention, particularly in the Sylhet region and other low-performing areas.
  • Specifically, now is the time for rethinking how to increase the number of anaesthetists trained and posted, including such innovative human resource solutions as public-private partnerships. One-year or six-month training on anaesthesiology could be made compulsory at graduation level, along with a better benefit package for rural postings. Compulsory rural service for two years can also be incorporated in the curriculum of medical graduation courses. An alternative solution could be training of nurse-midwives to give anaesthesia to overcome the hurdles.
  • Nurse-midwives are key to improved maternal health. The number of sanctioned posts for nurses should be increased with adequate training in maternal and newbnatal health, and an innovative marketing campaign should be initiated to ensure that more from low-performing districts join the profession. The nurses could be pivotal to provision of normal birthing care in rural UHCs. We recommend upgradation of their status and benefits within the current organizational and policy structure. Nurse-midwives for Sylhet should be drawn from that area given the language constraints. Social mobilization is needed for popularizing the nursing profession. Also, more in-depth studies are recommended to overcome hurdles with the nurses.
  • The one-year training of medical doctors in anaesthesia and obstetrics should be continued with efforts for better retention in rural areas of the Sylhet region after training. How to decrease the interference with postings remains unclear, but a human-resource plan with career paths could be a first step in this process of establishing uniformity and more transparency.
- Our data suggest that there is a need to train all categories of EOC service providers on evidence-based techniques and a supportive supervisory monitoring system be implemented.
- It is possible to monitor the quality of maternal healthcare, and monitoring should be incorporated in the routine management information system of the MoHFW. Private-sector facilities need to be included under this quality-monitoring systems.
- There is a need to strengthen the health system with adequate decentralization, devolution, and delegation of authority. To overcome all the constraints explored, a strong and vibrant health system is essential. Without good governance and stewardship, weaknesses of the prevailing health systems are difficult to address. Health managers need proper management training and an enabling environment, including political commitment, for better implementation of designed interventions.
- The context of care is important to consider in organizing maternity-care services. Regional variations in response to maternal care are obvious, and these variations require different plans and responses. While the push-system of management has its strengths, special strategies for improving the response in the low-performing areas is much needed.

[*Calculations*: We used the 2001 census data and a birth rate of 2.0% for Khulna and 2.06% for the Sylhet for calculation of the projected population and expected number of births (including stillbirths) for the calculation of need of human resources for maternal health as per the WHO 2005 estimation of 4 doctors and 20 nurse-midwives for every 3,500 births].
